# Adsorption Performance of Heavy Metal Ions under Multifactorial Conditions by Synthesized Organic-Inorganic Hybrid Membranes

**DOI:** 10.3390/membranes13050531

**Published:** 2023-05-19

**Authors:** Chaoqun Wu, Jiuhan Zheng, Limei Han

**Affiliations:** 1 Shanghai Civil Aviation College, 1 Longhua West Road, Shanghai 200232, China; 2 State Key Laboratory of Molecular Engineering of Polymers, Department of Macromolecular Science, Fudan University, 2005 Songhu Road, Shanghai 200438, China; 3 School of Pharmacy, Fudan University, Shanghai 201203, China

**Keywords:** organic-inorganic hybrid membrane, sol-gel method, epoxide ring opening, heavy metal cations, wastewater treatment

## Abstract

A series of hybridized charged membrane materials containing carboxyl and silyl groups were prepared via the epoxy ring-opening reaction and sol–gel methods using 3-glycidoxypropyltrimethoxysilane (WD-60) and polyethylene glycol 6000 (PEG-6000) as raw materials and DMF as a solvent. Scanning electron microscopy (SEM), fourier transform infrared spectroscopy (FTIR), and thermal gravimetric analyzer/differential scanning calorimetry (TGA/DSC) analysis showed that the heat resistance of the polymerized materials could reach over 300 °C after hybridization. A comparison of the results of heavy metal lead and copper ions’ adsorption tests on the materials at different times, temperatures, pHs, and concentrations showed that the hybridized membrane materials have good adsorption effects on heavy metals and better adsorption effects on lead ions. The maximum capacity obtained from optimized conditions for Cu^2+^ and Pb^2+^ ions were 0.331 and 5.012 mmol/g. The experiments proved that this material is indeed a new environmentally friendly, energy-saving, high-efficiency material. Moreover, their adsorptions for Cu^2+^ and Pb^2+^ ions will be evaluated as a model for the separation and recovery of heavy metal ions from wastewater.

## 1. Introduction

With the rapid development of the global economy, mankind’s best efforts to exploit the Earth’s environmental resources have also made our living environment increasingly harsh [1,2,3]. Heavy metal cations and certain toxic anions can cause serious pollution and hazards in the water environment, affecting fisheries and agriculture directly, and these ions are either directly or indirectly harmful to human health. For example, a single dose of 60–l00 mg/kg body weight of lead will stimulate the digestive system, causing abdominal pain, nausea, vomiting, and gastroenteritis, and a long-term overdose can lead to liver cirrhosis. Membrane separation is a new separation technology that emerged in the early 20th century and became increasingly popular after the 1960s. Membrane separation technology has been widely used in food, medicine, biology, environmental protection, the chemical industry, metallurgy, energy, petroleum, water treatment [4], electronics, bionics, and other fields, has produced huge economic and social benefits, and has become one of the most important methods in separation science today. Nowadays, membrane separation technology is widely used in water treatment [5,6] and the chemical, biological, and pharmaceutical fields at an industrial scale, showing its broad application prospects. Polyethylene glycol (PEG) is an excellent biodegradable and water-soluble biopolymer. Due to its environmentally friendly properties, PEG and its derivatives have been used extensively in the manufacture of PEG-based polymeric membranes or materials for various industrial applications. Although these membranes show some favorable properties, such as structural flexibility and excellent film-forming properties, they also show disadvantages, such as low mechanical strength and thermal stability. Therefore, improving their mechanical strength and thermal stability has turned out to be a key consideration in membrane preparation [7,8,9]. First of all, membrane separation is an efficient process that can achieve high purity. In addition, the membrane does not undergo phase change during the separation process, so the energy consumption is low. Secondly, membrane separation is usually carried out at room temperature, which is particularly suitable for handling heat-sensitive materials, and the separation equipment itself has no moving parts, making it highly reliable and easy to operate and maintain, which has very important theoretical and practical significance for the study of membrane separation technology to treat and recover heavy metal ions in order to reduce hazards. The performance of the membrane material is one of the most critical factors affecting the separation effect, so improving or enhancing the performance of the membrane material has become one of the core issues of membrane separation technology research.

Organic-inorganic hybrid materials are a new field of composite materials that combine many excellent properties of organic and inorganic materials, such as the low density, good viscoelasticity, high toughness, and good processability of organic materials, and the high hardness, high modulus of elasticity, high strength, better light transmission, and high refractive index of inorganic materials [10,11,12,13]. Organic-inorganic hybrid membrane materials combine the excellent properties of both organic and inorganic components, and their rapid development provides new ideas for the design and development of membrane materials [14,15,16,17,18]. In 1985, Schmidt H successfully prepared a class of amorphous solids using organically modified alkoxysilanes R′_n_Si(OR)_4−n_ (n = 1–3, R is alkyl and R′ is an organic short chain) as precursors via a sol-gel reaction [19]. Wilkes G. L. et al. condensed low-molecular-weight (500–1700) polydimethylsiloxanes with hydroxyl groups at the end groups (PDMS) and tetraethoxysilane (TEOS) in a sol-gel reaction under acidic conditions. During the sol-gel reaction, the Si-OH groups at the chain end of PDMS and TEOS undergo condensation to form Si-O-Si bonds so that the PDMS and inorganic SiO_2_ components are bonded together through covalent bonds, thus successfully preparing a PDMS-SiO_2_ system of organic-inorganic hybrid materials. The inorganic SiO_2_ network in these hybrids is bonded to polymer molecular chains, which Wilkes et al. named “ceramic polymers (creamers)” [20]. Following Schmidt and Wilkes, a number of researchers have explored organic-inorganic hybrids and have prepared them by various methods (e.g., sol-gel method [21], partial pyrolysis of organic polymers [22], nanotechnology copolymerization [23], chemical vapor deposition (CVD), etc.) [24,25,26]. To produce materials containing a variety of organic components, such as alkyl, phenyl, polystyrene, epoxy, polyether, and poly(ethylene glycol), epoxy resins, polyethers, polyimides, aminopropyl, aminophenyl, polymethyl methacrylate, and inorganic components, such as SiO_2_, Al_2_O_3_, and TiO_2_ have been combined with each other, and reviews of these materials have been published [27,28].

In recent years, sol-gel technology has developed rapidly in the preparation of organic-inorganic hybrid materials, and many papers have summarized it. [29]. The principle of the sol-gel method for the preparation of hybrid materials involves using inorganic compounds of metals as precursors, which are hydrolytically depolymerized, dehydrated and condensed to form inorganic networks [30], and adding organic substances or organic monomers with a common solvent to the inorganic substances to the inorganic sol to form inorganic-organic hybrid materials via condensation gelation. In recent years, many researchers have prepared a series of inorganic-organic hybrid membranes using inorganic and organic hybrid materials [31], which can not only significantly improve the thermal stability and mechanical strength of the materials and membranes, but can also use electrostatic effects to separate and adsorb heavy metal ions in industrial waste; thus, they have broad application prospects in wastewater treatment and environmental protection.

In this paper, a novel negatively charged siloxane hybrid membrane material is prepared by epoxy ring opening and sol-gel reactions to achieve the efficient and optimized separation of heavy metals at the molecular level. For this study, we chose Pb^2+^ and Cu^2+^ ions as typical adsorbed species because of their water polluting properties. The aim of this study is to optimize the molar ratio of organic and inorganic components, which is the most important factor affecting the degree of thermal stability and the separation effect. On this basis, the membrane material’s heavy metal ion adsorption properties were then investigated. The best adsorption pH, adsorption temperature, Cu^2+^/Pb^2+^ concentration, and adsorption time were identified, respectively, via the single variable method.

## 2. Materials and Methods

### 2.1. Materials

Silane coupler WD-60 (3-Glycidoxypropyltrimethoxysilane, 99%) was purchased from WD Silicone New Material Corporation (Hubei, China). Polyethylene glycol 6000 (PEG-6000), N,N-Dimethylformamide (DMF, 99.8%), maleic anhydride (99%), Cu(NO_3_)_2_, KI solution (10%, *w*/*w*), K_2_Cr_2_O_7_ (reference substance, dried at 140 °C for 2 h), Na_2_S_2_O_3_·5H_2_O solid, Na_2_CO_3_ solid, sulfuric acid solution (0.1 mol/L), hydrochloric acid solution (0.1 mol/L), nitric acid (0.1 mol/L), glacial acetic acid, sodium acetate, starch solution (0.5%, *v*/*v*), Pb(NO_3_)_2_, disodium ethylenediaminetetra acetate (EDTA), dimethoate orange indicator (0.2%, *w*/*w*), and methyltetramine buffer solution (20%, *w*/*w*) were purchased from Sinopharm Group Chemical Reagent Corporation (Shanghai, China). All chemicals were used as received unless otherwise stated.

### 2.2. Preparation of Hybrid Membranes

This class of hybrid membrane materials was prepared using N,N-dimethylformamide (DMF) as the solvent, and charged hybrid membrane materials containing acidic groups were prepared via the epoxy ring opening reaction and sol-gel methods. Four different ratios of materials were designed as presented in Table 1.

We started the DF-101S magnetic stirrer with a stirrer (900 rpm), thermometer, and heating device, increased its temperature to 80 °C, added 6.00 g of polyethylene glycol 6000 (PEG-6000) to the beaker, and then added 20 mL of DMF. We placed the beaker in the magnetic stirrer with heat collection in a water bath to dissolve it and then waited for it to cool for a while before using it. After adding 1.00 g of maleic acid and dissolving it in a water bath, we added an equal amount of WD-60 and heated the mixture in a DF-101S magnetic stirrer for 8 h. After the reaction was complete, the mixture was extracted and cooled to room temperature (27 °C). Then, we used a glass rod to coat it evenly on a Teflon board. We made sure to remove any air bubbles from the solution before coating, and we pumped it with a vacuum pump if required. We left it for 2–3 days at room temperature. After the material had been produced, we baked it for one day in a constant-temperature oven at 35 °C initially, then raised the temperature by 5 °C every other day. At 50 °C, the material was fully dry (usually 5–6 days). The samples were put in self-sealing bags and stored for testing after they had dried completely.

### 2.3. Membrane Characterization

To examine the microstructures, surface and cross-sectional morphology of the membranes, SEM (400 FEG, FEI) was performed. FTIR spectra were recorded on a Shimadzu FTIR-8400S spectrophotometer to determine the chemical compositions of the membranes. Solid samples were subjected to the KBr tablet method. All infrared spectra were accumulated 32 times with a 4 cm^−1^ resolution. The samples were analyzed thermally using a Netzsch STA 409 PC/PG integrated TGA/DSC (differential scanning calorimetry) thermal analyzer protected by nitrogen with a ramp-up rate of 10 °C/min; the temperature was increased from room temperature to 500 °C.

### 2.4. Adsorption Experiment

The adsorption experiment of organic-inorganic hybrid membranes for Cu^2+^/Pb^2+^ ions was conducted in a similar way as described in the previous articles [32,33,34], in which Cu^2+^/Pb^2+^ ions were used as the adsorption medium [35]. The detailed test procedure can be described in the Supplementary Materials (File S1). The adsorption capacity (*q*_Cu^2+^_) of Cu^2+^ ions (mmol/g) is calculated as follows (Equation (1)):

(1) $$q_{{\mathrm{Cu}}^{2+}}=\frac{(C_{0}-C_{R})V}{W}$$

where *V* is the volume of aqueous Cu(NO_3_)_2_ solution (mL), *C*_0_ and *C_R_* are the concentrations of initial and remaining Cu(NO_3_)_2_, respectively (mol/L), and *W* is the weight of hybrid membrane (g). The adsorption capacity (*q*_Pb^2+^_) of Pb^2+^ ions (mmol/g) can also be calculated by Equation (1), where *V* should be changed to the volume of aqueous Pb(NO_3_)_2_ solution (mL), *C*_0_ and *C_R_* are the concentrations of initial and remaining Pb(NO_3_)_2_, respectively (mol/L), and *W* is the weight of hybrid membrane (g) (File S2).

**Figure 1:**
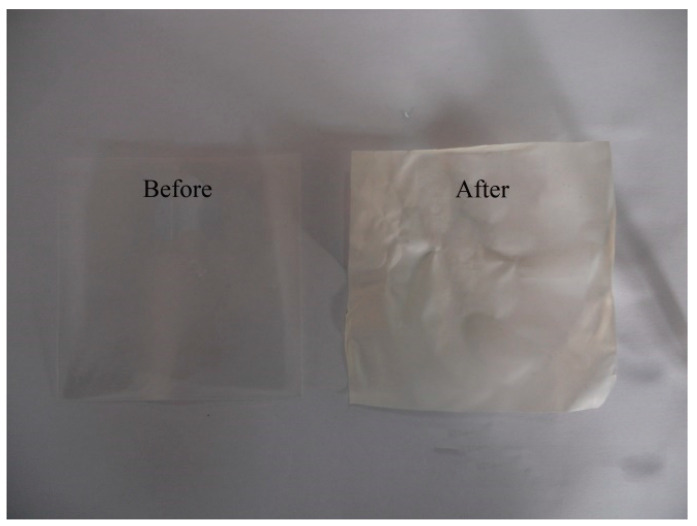
Macrographs of membrane material before (**left**) and after (**right**) adsorption.

The effect of adsorption pH, adsorption temperature, Cu^2+^/Pb^2+^ concentration, and adsorption time were performed to determine optimal conditions. The macroscopic morphology of the prepared hybrid membrane materials before and after adsorption can be visualized in Figure 1. 

## 3. Results and Discussion

### 3.1. Characterization of Hybrid Membranes

**Figure 2:**
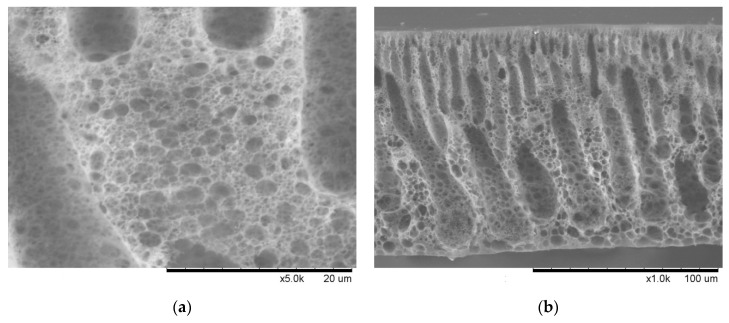
(**a**) Surface structure of PEG/Si-O-Si organic-inorganic hybrid materials; (**b**) Cross-sectional morphology of PEG/Si-O-Si organic-inorganic hybrid materials.

The microscopic surface structure and cross-sectional morphology of the organic-inorganic hybrid membrane were clearly characterized in scanning electron microscopy (SEM) micrographs. As shown in Figure 2, it is obvious to find that the surface of the hybrid membrane (sample A) exhibits a larger pore-size structure [36].

**Figure 3:**
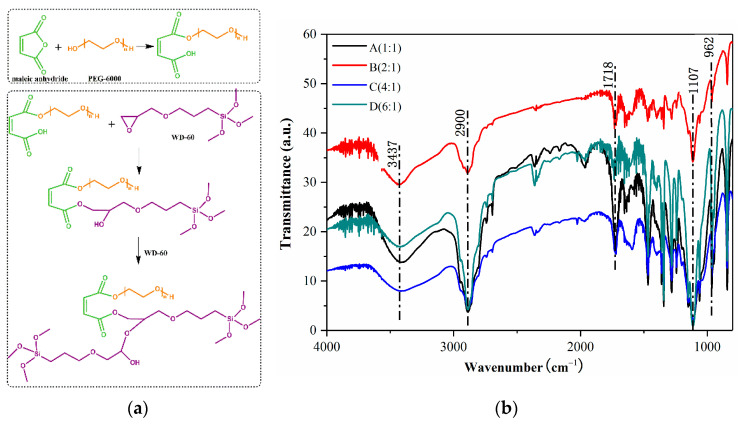
(**a**) Synthesis of PEG/Si-O-Si organic-inorganic hybrid materials; (**b**) FTIR spectra of the hybrid membrane materials (A, B, C, and D).

PEG/Si-O-Si organic-inorganic hybrid materials were synthesized via an epoxy ring-opening reaction, as shown in Figure 3a. Figure 3b illustrates the FTIR spectra of the hybrid membrane materials, which all exhibited Si-O-Si stretching vibration absorption peaks at 1107 cm*^−^*^1^; in the range of 2700–2900 cm*^−^*^1^, there were larger broad peaks, primarily methylene and methylene absorption peaks, demonstrating that the carbon chain was not broken in the reaction; 2900 cm*^−^*^1^ was the asymmetric stretching vibration peak of –CH_2_; 3437 cm*^−^*^1^ was the –OH stretching and bending vibration peak; and the characteristic absorption peak of –COOH appeared at 1718 cm*^−^*^1^, and the peak increased as the ratio increased, indicating that the content of –COOH also increased [19,20,28]. In addition, the spectra of the other three ratios showed an increase with the amount of WD-60. The characteristic absorption peak for epoxy was near 962 cm*^−^*^1^, showing that there were epoxy group residues in the system, but the peak area was tiny, indicating that there were fewer epoxy residues in the system.

**Figure 4:**
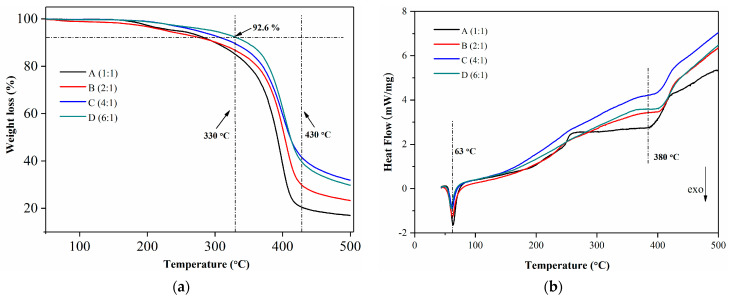
(**a**) TGA curves for different ratios of silica-based hybrid membranes; (**b**) DSC curves for different ratios of silica-based hybrid membranes.

Figure 4a shows TGA curves with different ratios of silica-based hybrid membranes. As can be seen from the figure, there were a total of three weight loss processes with an onset temperature of 44 °C. The first weight loss process occurred between 44 °C and 300 °C, with a mass reduction of approximately 10% of the total initial mass of the specimen. Significant weight loss occurred between 300 and 440 °C, with a mass reduction of approximately 50% of the initial total mass of the specimen, indicating that the thermal stability of the hybrid material could reach above 300 °C. With an increase in inorganic WD-60, the thermal stability of the hybrid material increased. The third, which occurred between 440 °C and 500 °C, was a plateau process with essentially constant mass, where an increase in the inorganic WD-60 meant that the amount of inorganic material remaining in the hybrid material also increased. Meanwhile, the weight loss beyond 330 °C can be ascribed to the further degradation of organic groups and the production of crystallized silica [35]. It can be seen from Figure 2a that the thermal stability of the hybridized membrane material is best when the ratio is 6:1; the weight loss around 330 °C was 7.4%. This sample, D, demonstrates a relatively higher compatibility between the inorganic and organic ingredients [35].

The DSC curves for the various ratios of the hybridized membrane materials are shown in Figure 4b. The glass transition temperature of the hybridized material, where the absorbing group exerted its heat, was around 63 °C, where a downward peak can be seen. The peak shifted to the right as the ratio rose at about 380 °C, showing that the stability of the material rises in step with the ratio.

### 3.2. Absorption Properties of Cu^2+^ by Hybrid Membrane Materials

**Figure 5:**
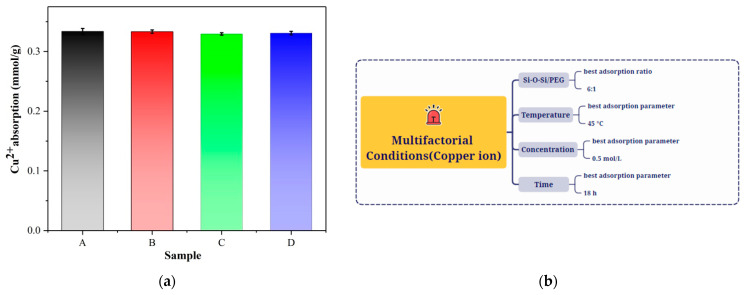
(**a**) Adsorption capacity of different samples (A, B, C, and D) for Cu^2+^ ions; (**b**) Cu^2+^ adsorption experiment with the hybrid membrane under multifactorial conditions.

Figure 5a demonstrates that there was no significant change of Cu^2+^ adsorption effect. As the amount of WD-60 increased, the adsorption effect slightly increased, and the *q*_Cu^2+^_ was about 0.33 mmol/g, which was three times higher than in previous studies (0.075 mmol/g) in Table 2. The Cu^2+^ adsorption schemes of the hybrid membranes (A, B, C, and D) under multifactorial conditions are shown in Figure 5b.

**Figure 6:**
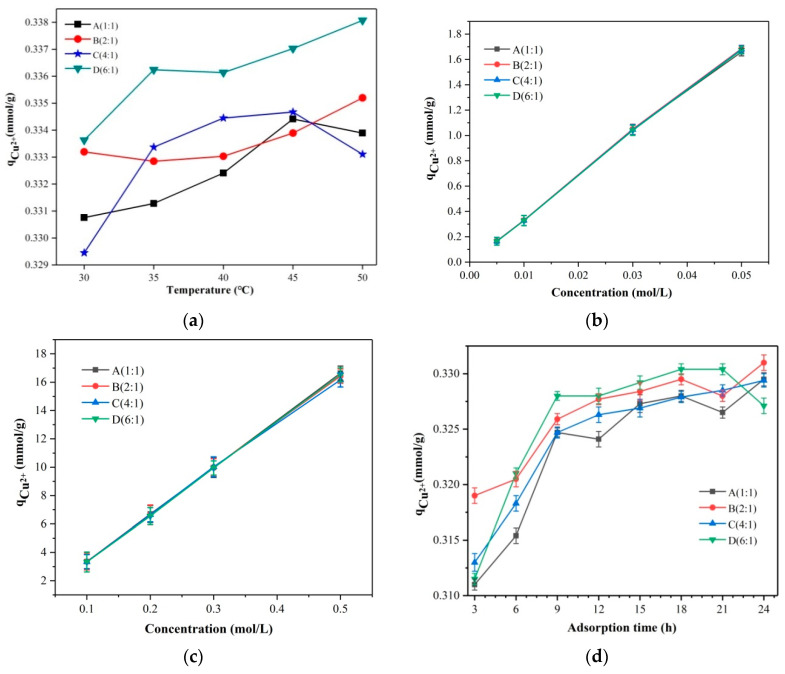
(**a**) Cu^2+^ ion adsorption curves of A, B, C, and D at different temperatures; (**b**) Adsorption curves of A, B, C, and D at different Cu^2+^ ion concentrations of 0.005 mol/L, 0.01 mol/L, 0.03 mol/L and 0.05 mol/L; (**c**) Adsorption curves of A, B, C, and D at different Cu^2+^ ion concentrations of 0.1 mol/L, 0.2 mol/L, 0.3 mol/L, and 0.5 mol/L; (**d**) Cu^2+^ ion adsorption curves of A, B, C, and D at different time.

The constant temperature vacuum drying oven was set at 30, 35, 40, 45, and 50 °C. The adsorption capacity of the hybrid membrane material for Cu^2+^ ions fluctuated with temperature, as can be seen in Figure 6a, with the best adsorption effect occurring at 45 °C. The optimal temperature for Cu^2+^ adsorption by the hybrid membrane material was therefore determined to be 45 °C. The same conclusion can be found in the previous articles for the zwitterionic hybrid adsorbents [32].

Different concentrations of 0.005 mol/L, 0.01 mol/L, 0.03 mol/L, 0.05 mol/L, 0.1 mol/L, 0.2 mol/L, 0.3 mol/L, and 0.5 mol/L of Cu(NO_3_)_2_ were prepared, and their pH was adjusted to pH = 4. The volume of Na_2_S_2_O_3_ consumed can be used to compute the amount of sample adsorbed. The adsorption of the membrane materials all increased with the concentration of Cu^2+^ ions, as can be seen in Figure 6b,c. The reason for this is that as the Cu(NO_3_)_2_ solution’s concentration increased, meaning that more Cu^2+^ ions engaged in ion exchange, which increased the amount of Cu^2+^ ions that the hybrid polymer was able to adsorb.

Figure 6d shows that the hybrid membrane material’s adsorption amount gradually increased over time and eventually tended to be constant, indicating saturation adsorption. This is because the ion exchange capacity of the hybrid material, which was correlated with adsorption, progressively rose over time and eventually approached saturation at the optimum adsorption duration. The equilibrium adsorption time was 9 h, at which time the membrane’s adsorption capacity was essentially saturated.

The material adsorbed Cu^2+^ ions very quickly, and Table 3 demonstrates that the amount of adsorption progressively increased with time and that the rate of adsorption was speedy, with the first 9 h accounting for 99.3% of the maximum adsorption of the entire process.

### 3.3. Absorption Properties of Pb^2+^ by Hybrid Membrane Materials

**Figure 7:**
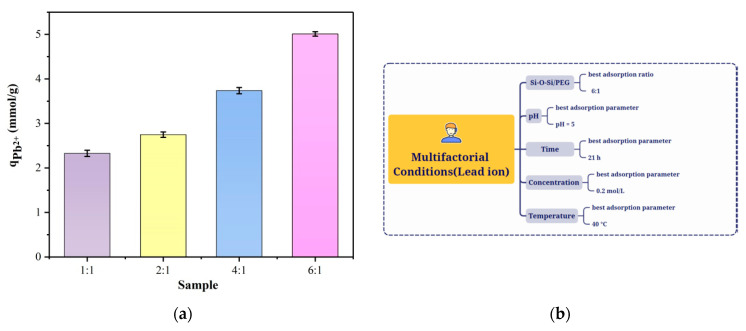
(**a**) Adsorption capacity of different samples (A, B, C, and D) for Pb^2+^ ions; (**b**) Pb^2+^ adsorption experiment by the hybrid membrane under multifactorial conditions.

The 6:1 material has the highest adsorption capacity of 5.012 mmol/g, as can be seen in Figure 7a. In the following experiments, we looked at the degree of Pb^2+^ ion adsorption for materials A, B, C, and D at various times, concentrations, and pH values (Figure 5b). As can be seen in Figure 5a, the adsorption capacity of the material for Pb^2+^ ions became stronger as the organic composition increased.

The theoretical explanation for this phenomenon is that the amount of adsorption of the hybrid material may be related both to the charging characteristics of the hybrid material and to the network structure formed by the hybrid material. Different structures also show different adsorption for different metal ions. For the adsorption of Pb^2+^ ions, which was mainly based on ion exchange, a large number of hydrogen ions were exchanged with Pb^2+^ ions in solution [14,19,28], so the larger the WD-60 ratio, the greater the amount of adsorption (Figure 7b).

**Figure 8:**
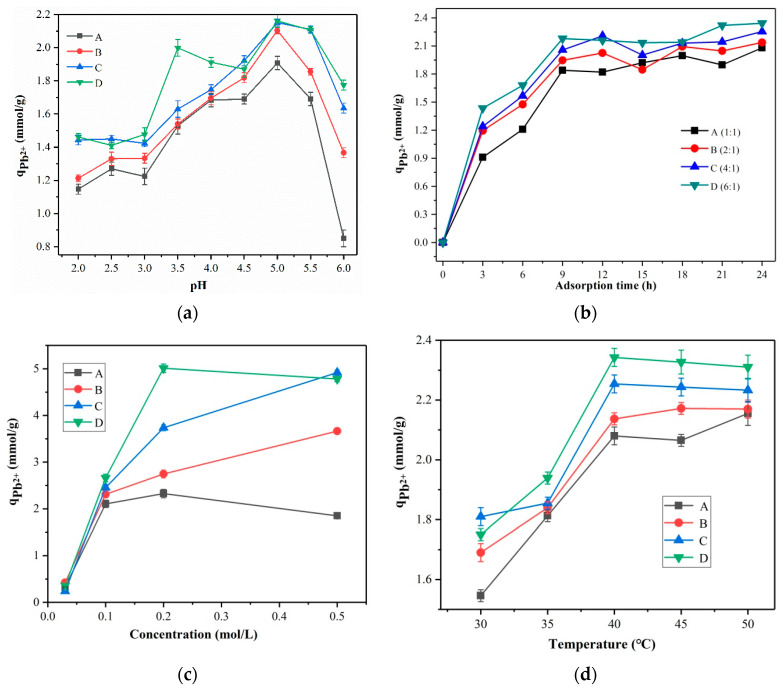
(**a**) Pb^2+^ ion adsorption curves of A, B, C, and D at different pHs; (**b**) Pb^2+^ ion adsorption curves of A, B, C, and D at different times; (**c**) Adsorption curves of A, B, C, and D at different Pb^2+^ ion concentrations of 0.03 mol/L, 0.1 mol/L, 0.2 mol/L and 0.5 mol/L; (**d**) Pb^2+^ ion adsorption curves of A, B, C, and D at different temperatures.

At a pH of 5, the four materials were best at adsorbing Pb^2+^. The optimal pH for Pb^2+^ adsorption by materials A, B, C, and D hybrids was therefore determined to be 5 (Figure 8a). The maximum adsorption capacity (*q*_Pb^2+^_) for the four materials was reached at 21 h. (Figure 8b). Because ion exchange adsorption was the primary cause of Pb^2+^ ion adsorption, the best adsorption effect of the hybrid material was obtained at a Pb^2+^ ion concentration 0.2 mol/L, as can be seen in Figure 8c. The adsorption performance of the hybrid material was subsequently improved by increasing the amount of WD-60 at various concentrations. The adsorption capability of the hybrid membrane material for Pb^2+^ ions fluctuated with temperature, as can be seen in Figure 8d, with the best adsorption occurring at 40 °C. Therefore, 40 °C is the ideal temperature for the hybrid membrane material to adsorb Pb^2+^ ions. The theoretical explanation to this phenomenon can be ascribed to the electrostatic attraction between Pb^2+^ ions and carboxylic groups on the polymer chains [36].

Commonly, when metal ions are adsorbed by an adsorbent, the metal ions transport from the solution through the interface between the solution and adsorbent into the pores of the network structure. To have an insight into the effect of intraparticle diffusion on adsorption rate, sample A–D can be calculated based on the relationship of the adsorption capacity and time, which usually is expressed as (Equation (2)) [32,36]:

(2) $$q_{t}=x_{i}+k_{p}t^{0.5}$$

where *q_t_* is the adsorbed amount (mmol/g) at time *t*, *k_p_* is the intraparticle diffusion rate constant, and *x_i_* is the intercept of the straight line, which is related to the boundary layer thickness. It is now well accepted that if the plot of *q_t_* vs. *t*^0.5^ gives a straight line, the adsorption process is solely controlled by intraparticle diffusion. If the data exhibit multilinear plots, however, two or more steps will influence the adsorption process [32,36].

**Figure 9:**
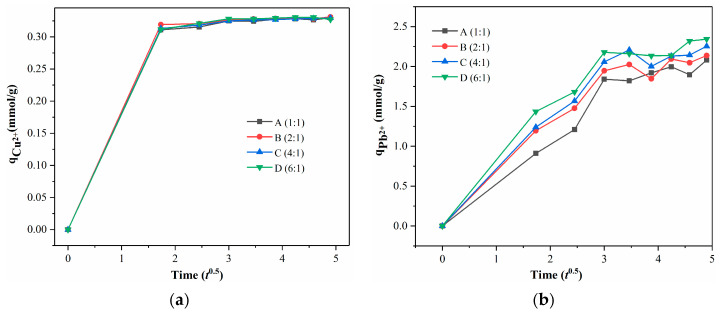
(**a**) Intraparticle diffusion curves for Cu^2+^ adsorption on samples A–D; (**b**) Intraparticle diffusion curves for Pb^2+^ adsorption on samples A–D.

Figure 9 illustrated the intraparticle diffusion curves for Cu^2+^ and Pb^2+^ ions adsorption in this case. As shown in Figure 9, it can be found that the adsorption of Cu^2+^ and Pb^2+^ ions on samples A–D is non-linear over the range of adsorption time and three steps are involved: the rapid interface diffusion from 0 to 3 h; subsequently, the intraparticle diffusion (increased slowly and approached equilibrium), suggesting that Cu^2+^ and Pb^2+^ adsorption on samples A–D is not governed by intraparticle diffusion; diffusion-controlled adsorption mechanisms might be the major process, as reported in other articles [32,36].

Table 4 below shows the adsorption capacity (*q*_Pb^2+^_) percentage of the first 9 h to 21 h (maximum adsorption capacity). Table 4 shows that the rate of adsorption was quick and that the amount of adsorption gradually increased over time, with the first 9 h accounting for a maximum of 93.0% of the entire process.

## 4. Conclusions

Using epoxy ring opening and sol-gel reactions, a new siloxane hybrid membrane material has been devised to enhance the heavy metal ion separation capabilities of organic-inorganic membranes under multifactorial reaction conditions. FTIR results proved that sol-gel technology was suitable for the preparation of organic-inorganic hybrid materials and the PEG and inorganic Si-O-Si components were bonded together through covalent bonds, thus successfully preparing a PEG/Si-O-Si system of ion-exchange membrane materials. TGA/DSC measurements revealed significantly improved thermal stability following the hybridization of these membranes, with the increased amount of inorganic material (WD-60) contributing to the molecular structural stability of the hybrid materials. The hybrid membrane material surfaces displayed high and efficient separation activity against Cu^2+^ and Pb^2+^ with varying degrees of heavy metal wastewater pollution. This study describes a low-cost but effective approach to preparing membrane separation surfaces and solves the problem of multifactorial reaction conditions and complicated operations from the perspective of industrial controllability. The best adsorption pH (Figure 5b), adsorption temperature, adsorption concentration, and adsorption time (Figure 7b) were found in turn via the single variable method, suggesting that these materials are likely to be widely used in water treatment and the chemical, biological, and pharmaceutical fields at an industrial scale with broad application prospects. In the practical condition, the membrane has to face the complicated feed solution. Future research will continue to investigate the microscopic properties of membrane materials to adapt to complex environments.

## Figures and Tables

**Table 1:** Proportion of raw materials for the preparation of hybrid membranes.

Sample	Ratio (x:y)	Amount of WD-60 (g)	Amount of PEG-6000 (g)	DMF Volume (mL)	Amount of Maleic Anhydride (g)
A	1:1	2.36	6.00	20	1.00
B	2:1	4.72	6.00	20	1.00
C	4:1	9.44	6.00	20	1.00
D	6:1	14.14	6.00	20	1.00

x:y indicates the molar ratio of WD-60 to PEG-6000.

**Table 2:** Comparison of Q_m_ values obtained from samples A–D with those of different types of sorbents reported in the literature.

Sorbent Type	*q*_Cu^2+^_ (mmol/g)	*q*_Pb^2+^_ (mmol/g)	Literature
zwitterionic hybrid copolymers	0.075	0.244	[32,33]
silica-based hybrid adsorbents	/	0.506	[34]
pyromellitic acid dianhydride (PMDA) hybrid adsorbents	/	6.379	[36]
negatively charged siloxane hybrid membrane	0.331	5.012	this work

**Table 3:** The proportion of Cu^2+^ adsorption in the first 9 h of the overall process.

Samples	Adsorption Rate (%)	Standard Deviation (%)
A	98.5	0.08
B	98.5	0.05
C	98.6	0.06
D	99.3	0.05

**Table 4:** Proportion of Pb^2+^ adsorption in the first 9 h of the overall process.

Samples	Adsorption Rate (%)	Standard Deviation (%)
A	88.5	0.3
B	91.1	0.2
C	91.3	0.3
D	93.0	0.4

## Data Availability

The data presented in this study are available on request from the corresponding author.

## Supplementary Materials

The following supporting information can be downloaded at: https://www.mdpi.com/article/10.3390/membranes13050531/s1, File S1: Determination of the adsorption capacity for Cu^2+^; File S2: Determination of the adsorption capacity for Pb^2+^.
