# Supplementary material for: Adsorption Performance of Heavy Metal Ions under Multifactorial Conditions by Synthesized Organic-Inorganic Hybrid Membranes
Source: Membranes (Basel). 2023 May 19;13(5):531. doi: 10.3390/membranes13050531 (PMC10224549; doi:10.3390/membranes13050531)
Supplement: Supplementary file 1 [file membranes-13-00531-s001.zip › membranes-2345190-supplementary.pdf]

membranes

Supplementary Materials

Adsorption Performance of Heavy Metal Ions under Multifactorial Conditions by Synthesized Organic-Inorganic Hybrid Membranes

Chaoqun Wu [1,*], Jiuhan Zheng [2] and Limei Han [3]

[1] Shanghai Civil Aviation College, 1 Longhua West Road, Shanghai 200232, China
[2] State Key Laboratory of Molecular Engineering of Polymers, Department of Macromolecular Science, Fudan University, 2005 Songhu Road, Shanghai 200438, China
[3] School of Pharmacy, Fudan University, Shanghai 201203, China
* Correspondence: wuchaoqun@shcac.edu.cn; Tel.: +86-187-1772-0095

File S1. Determination of the adsorption capacity for Cu^{2+}

In a weakly acidic solution (pH = 3–4), Cu^{2+} can be reduced to CuI by KI with the reaction equation:

$$2Cu^{2+} + 4I^{-} = 2CuI\downarrow + I_2 \tag{1}$$

This is a reversible reaction due to the relatively low solubility of CuI. In the presence of excess KI, the reaction proceeds quantitatively to the right, and the precipitated I_2 is titrated with $Na_2S_2O_3$ standard solution using starch as an indicator to indirectly measure the amount of copper in the solution. The reaction equation is:

$$I_2 + 2S_2O_3^{2-} = 2I^{-} + S_4O_6^{2} \tag{2}$$

We pipetted 40 mL of 0.01 mol/L copper nitrate solution (pH = 3.5) into a 100 mL conical flask, took 1.00 g of hybrid membranes in the conical flask, placed them all in a vacuum oven at a constant temperature of 40 °C for 24 h, collected the filtrate in an extraction flask after reaching the specified time, and then transferred the filtrate into a conical flask (labeled 1) and placed it on an electronic balance to weigh it (we added a quantity of distilled water so that they weighed 90.00 g each time). We took a dry conical flask (labeled 2) and placed it on the electronic balance and set the balance to zero. Using a rubber-tipped dropper, we took the liquid from the beaker (labeled 1) and added it to the conical flask (labeled 2) so that the balance read 30.00 g. We took another dry conical flask (labeled 3) and placed it on the electronic balance and set the balance to zero. Using a rubber-tipped burette, we added the liquid from the beaker (labeled 1) to the conical flask (labelled 3) so that the electronic balance read 30.00 g. We added 30 mL of deionized water to the conical flask, then added 40 mL of deionized water to all three conical flasks and adjusted the pH of the solution with a buffer solution of acetic acid and sodium acetate (pH = 3.5). Then, 10 mL of a 10% mass fraction of the solution was titrated with the calibrated sodium thiosulphate solution, and the solution turned from coffee to bright yellow. When the solution appeared beige with bright yellow, we added 5 drops of starch indicator with a mass fraction of 5%, and once the solution turned blue, we continued to add sodium thiosulphate drop by drop until the solution turned beige. After standing for one minute, if the color of the solution did not change, this was considered the end point. We then recorded the volume of the sodium thiosulphate solution consumed at the end point.

File S2. Determination of the adsorption capacity for Pb^{2+}

We prepared a standard solution of lead nitrate (pH = 4) with a concentration of 0.2 mol/L. We took 1.0 g of each sample, placed it in a 150 mL conical flask, pipetted 40 mL

of lead nitrate solution into it, and placed it in a vacuum drying oven at a constant temperature of 30 °C to allow it to adsorb for 24 hours. We then filtered the sample, collected the filtrate, and titrated it with EDTA standard solution. The end point was reached when the EDTA was titrated to bright yellow or purplish red. The volume of EDTA consumed was used to calculate the amount adsorbed by each sample.
